# Nurses Training and Capacitation for Palliative Care in Emergency Units: A Systematic Review

**DOI:** 10.3390/medicina56120648

**Published:** 2020-11-26

**Authors:** Sonia Ortega Romero, Almudena Velando-Soriano, José Luis Romero-Bejar, Keyla Vargas-Román, Luis Albendín-García, Nora Suleiman-Martos, Guillermo Arturo Cañadas-De la Fuente

**Affiliations:** 1 Andalusian Health Service, San Cecilio University Hospital, Avenida de la Ilustración s/n, 18016 Granada, Spain; sortegaromero@gmail.com; 2 Granada-Metropolitan District, Andalusian Health Service, Avenida del Sur 11, 18014 Granada, Spain; srtavelando@correo.ugr.es (A.V.-S.); lualbgar1979@ugr.es (L.A.-G.); 3 Department of Statistics and Operational Research, University of Granada, 18071 Granada, Spain; 4 Faculty of Psychology, University of Granada, Campus Universitario de Cartuja s/n, 18071 Granada, Spain; keyvarom@ugr.es; 5 Faculty of Health Sciences, University of Granada, Cortadura del Valle s/n, 51001 Ceuta, Spain; norasm@ugr.es; 6 Faculty of Health Sciences, University of Granada, Avenida de la Ilustración N. 60, 18016 Granada, Spain; gacf@ugr.es

**Keywords:** emergencies, meta-analysis, nursing care, palliative care, terminal illness

## Abstract

*Background and objectives:* Palliative care (PC) prevents and alleviates patients´ suffering to improve their quality of life in their last days. In recent years, there has been an increase in visits to the emergency services (ES) by patients who may need this type of care. The aims were to describe the training and capacitation of nurses from ES in PC. Accordingly, a systematic review was performed. *Materials and Methods:* Medline, Scopus, and Cumulative Index to Nursing and Allied Health Literature (CINAHL) databases were used. The search equation was “Palliative care and nursing care and emergency room”. A total of 12 studies were selected. *Results:* The studies agree on the need for training professionals in PC to provide a higher quality care, better identification of patient needs and to avoid unnecessary invasive processes. Similarly, the implementation of a collaborative model between ES and PC, the existence of a PC specialized team in the ES or proper palliative care at home correspond to a decrease in emergency visits, a lower number of hospitalizations or days admitted, and a decrease in hospital deaths. *Conclusions:* The development of PC in the different areas of patient care is necessary. Better palliative care leads to a lower frequency of ES by terminal patients, which has a positive impact on their quality of life. Access to PC from the emergency unit should be one of the priority health objectives due to increment in the aged population susceptible to this type of care.

## 1. Introduction

According to the World Health Organization (WHO) and the Spanish society of palliative care (PC) definition, a terminal disease is one that does not have a specific curative treatment or with the ability to delay evolution and that leads to death in a variable time, which generally is less than six months. It is progressive; causes intense, multifactorial, changing symptoms; and entails great suffering (physical, psychological, social and existential) for the patient and their family [1,2].

In recent decades, along with the progressive aging of the world population, we are seeing a gradual increase in the prevalence of some chronic diseases. According to the statistical data of the continuous Register of the National Institute of Statistics (INS) in January 2020, in Spain, there were 9,217,464 people over the age of 65, accounting for 19.4% of the total population. A clear increase is reflected taking into account that, in the register of January 2018 there were 8,908,151 elderly people, or 19.1% of the population [3]. The European Union countries with the highest number of older people in 2017 were Germany (17.5 million), Italy (13.5 million), France (12.9 million), United Kingdom (11.9 million), and Spain (8.8 million) [3,4]. The percentage of people aged 65 and over has been increasing in recent years and will continue to increase, with the increase in the percentage of the population over 80 expected to be even greater. This fact is what has been called "aging of aging", an eminently feminine phenomenon, since the older groups will be composed mostly of women [5]. Between 2015 and 2050, the proportion of people over 60 will increase from 605 to 2000 million, representing an increase from 12 to 22% [1].

The improvement of the care of patients in advanced and terminal phase is one of the challenges posed by the Spanish Health that dates back to the 90s. Measures to achieve this must include: implementation of specific resources; improvement of care in existing resources such as primary care, hospitals, and long-stay centers; training of professionals; and education of society and its participation through volunteering [1,6]. Facilitating the use of opioid analgesics is also vital for improved care, a measure unanimously recommended by all experts and the WHO in the context of PC. In Terminal situations, the goal of medical care is not to “cure” but to “care” for the patient, despite the persistence and irreversible progression of the disease. It is about providing the highest quality of life until death happens [1,6].

In recent years, there has been an increase in the number of visits of patients who may need PC in the hospitals’ emergency services (ES). This is due to the exacerbation of chronic diseases in the elderly population that has occurred in recent decades [6,7]. However, there are differences in the approach and management of palliative care patients between emergency units and palliative care units [8] and sometimes there is a failure to address goals of care of these patients [9]. Thus, there is a knowledge gap about PC in ES [9] and it is important to analyze emergency nurses’ preparation for this kind of care. 

There are still no definitive models for the implementation of PC or specialized personnel for this in the ES, which may be due to the shortage of qualified personnel, the increased demand, and the care system in the ES, which is designed to manage the urgent signs and symptoms, not the disease as a whole. On the contrary, the PC team includes comprehensive and active patient care [8].

It is important for nurses working in ES to know how to identify the different symptoms that a person receiving palliative care may have in order to act, together with others healthcare professionals, as soon as possible effectively. Therefore, bringing this type of care to patients who need it in the ES has become an interesting area of study.

The main objectives of the study were: (a) to describe the training, capacitation, and interventions of registered nurses that works in the ES regarding PC, and (b) to describe the influence of PC in visits to ES and to analyze the PC access model when attending emergency services.

## 2. Materials and Methods

A systematic literature review was done.

### 2.1. Information Sources and Search Criteria

Medline, Scopus, and CINAHL databases were used, including articles published until March 2020. The search equation used was “Palliative care AND nursing care AND emergency room”. The descriptors of the search equation were taken from the thesaurus Medical Subject Headings (MeSH). The search was conducted in March 2020, including studies published until that month.

We included quantitative studies on the performance of PC in the ES or that were related to our study, published in English or Spanish, without restriction by year of publication. Qualitative studies, doctoral theses, and systematic reviews were excluded.

### 2.2. Studies Selection Process and Evidence Level

**Figure 1:**
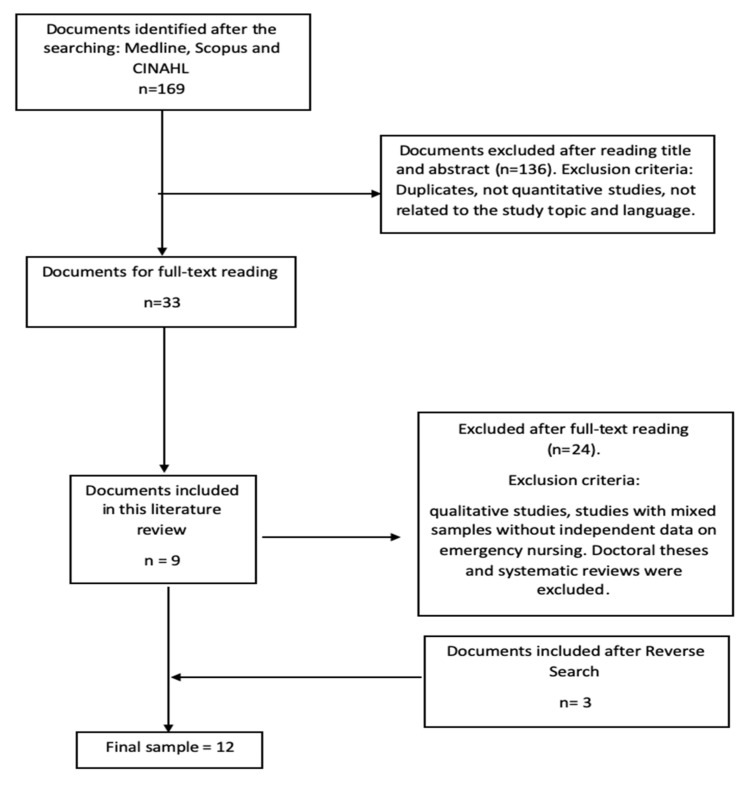
Flow chart of bibliographic search.

The selection was made in 4 phases. First, the title and abstract of the articles were read. The full text was then read and after that a reverse search (searching in the references of the selected studies) was done with the included studies to locate as many studies as possible. Finally, the critical reading of the studies was done to evaluate possible biases in the methodology. This process is shown in Figure 1.

To assess the quality of the studies included in the review, the Oxford Centre for Evidence-Based Medicine (OCEBM) levels of evidence and degrees of recommendation were followed [10]. The evidence level varies from 1a (the highest) to 5 (the lowest), and it is selected depending on the study method and bias. The grade of recommendation can be A (including levels of evidence 1a, 1b and 1c), B (including levels of evidence 2a, 2b, 2c, 3a and 3b), C (including level of evidence 4), and D (including level of evidence 5).

### 2.3. Data Collection and Data Analysis

Two types of variables were collected: (a) Variables about the characteristics of the sample: Year of publication, country of study, language of publication (Spanish vs. English), study design and aim. (b) variables on the application of palliative care in the ES: Need for a nurse/palliative care team in the emergency department, main symptoms that palliative care patients present when they arrive at the emergency room, main diseases that require palliative care, and training of professionals in terms of knowledge and application of palliative care procedures in ES. The data collection was performed using a notebook for each study including the mentioned variables. A descriptive analysis of the selected studies was done for the systematic review.

## 3. Results

### 3.1. Studies Selection and Characteristics of the Studies

A total of 169 studies were obtained after the search, obtaining a final sample of *n* = 12, after eliminating duplicates, applying the inclusion and exclusion criteria, and doing the reverse search. The selection process is shown in Figure 1.

Of the 12 studies included in the review, five were cross-sectional studies, five were cohort studies and two were clinical studies. This information is shown in Table 1.

### 3.2. Nurses’ Training, Capacitation and Interventions in Palliative Care Patients that Visit Emergency Services

#### 3.2.1. Emergency Nurses Interventions for Terminal Patients

The author Pereira [11] stated that the most common procedures in terminal patients performed by nurses in the ES were the channeling of peripheral venous pathways (95.2%) for the administration of intravenous therapy in 98.8% of cases or for the administration of rescue medication for symptomatic pain control (66.3%), with opioids in 33.7% of cases and neuroleptics or benzodiazepines in 19.3% and 13.3% of cases. It also describes the use of the subcutaneous route, the first-choice route in terminal patients on palliative medication, but only for the administration of heparin (32.5%) and insulin (21.7%). In addition to this practice, other interventions performed by nurses when the patient’s death was imminent are highlighted: resuscitation maneuvers (26.3%); aspiration of secretions (83.1%), cardiac and vital signs monitoring (98%), blood glucose monitoring (92.8%), electrocardiogram (49.4%), or the use of complementary oxygen (83.1%) [11,14,16].

With respect to the assessment and skin care, in the 88% of the cases an assessment of the skin using the scale of Braden was done and in the 68.7% of the cases dressings were used and the skin was protected in a 28.9% [10]. However, not all data on the care were so positive, because only 2.4% of the professionals pay special attention to the comprehensive care of the patient and, in 97.6% of the cases, basic care such as oral hygiene were not registered and 54.6% of the hygiene (bathing, changing clothes...) were not care registered [11].

#### 3.2.2. Emergency Nurses Training and Capacitation for PC

Another aspect to be highlighted is the perception of PC training in the ES. According to the authors Shearer et al. [16], the confidence in the provision of PC was lower in the nursing staff than the physicians. However, both professionals showed educational needs, with deficiencies in the area of communication and the ethical issues that arise in the context of PC [15]. However, according to another study, also conducted in Australia, all staff reported that they felt confident with the management of symptoms and signs in patients with PC needs [16].

Some studies stated that medical staff felt safer with communication oriented to decision-making, while nursing staff preferred the performance of invasive/non-invasive techniques [15,16]. Communication skills at the end of life (giving bad news, talking to the family, discussing prognosis, or discussing treatment options) and ethical issues (advanced care directives and building patient decision-making capacity) were the two most requested areas for training [17].

In an intervention study done by Weng et al. [16] with 94 emergency nurses implementing a new PC model that included training and educational programs it was obtained a clear increase in the capacity of the staff to perform PC; before the educational intervention only 64 emergency nurses were considered competent to perform PC compared to 90 emergency nurses after the training. This was also reflected in the telephone consultations of these nurses with the PC area for the resolution of complications. Before the intervention, no consultations were made as patients were not considered for PC and after the intervention increased to 19 consultations per month, so the ability to know when and how to consult the PC team increased by 95% in the after the intervention [15].

In the first study conducted by Shearer et al. [17], only nine of the participants correctly identified these causes, while in the study by Russ et al. [18], only two of the sixty-five respondents correctly identified them. According to both studies, which reflect data from the Statistical Office of Australia (ABS), the top five causes of death classified as most appropriate for receiving PC were: pancreatic cancer (72.30%); trachea, bronchial, and lung cancer, including COPD (70.16%), sigmoid colon, rectum or anus cancer (60%), breast cancer (44.61%), and dementia and Alzheimer’s (43.7%).

In the study by Russ et al. [18], the medical staff disagreed more with the statement “initial discussions about the end-of-life care should be deferred until there are no more curative treatments available” compared to the nursing staff who gave more priority to complete pain relief, patient well-being and identification of psychosocial problems that cause distress in these patients [17,18].

### 3.3. Access Model to Palliative Care in ES and Influence of PC Nurses in the ES

#### 3.3.1. Access to Palliative Care. Emergency Department-Palliative Service (ED-PALS) Care Model

Koh et al. [14], advocates a tripartite model of collaboration in PC between the emergency department, the PC unit and hospitalization, allowing earlier access to PC units. This study brought together a total of 340 cancer patients who were referred to the ES. According to this model, the emergency room physician should contact PC professionals to evaluate the patient; once evaluated, they could be referred to their home for outpatient PC, to hospitalization (41%) or directly to the PC unit (35%). A “comfort area” was designed for patients whose estimated survival was less than 48 h (6%), where they could have greater privacy with their family and relatives [15]. One of the main limitations of this study was the inability to contact the PC service outside working hours (8.00–17.00 h) [14,19]. This limitation was solved by enabling telephone calls in the most severe cases and if there were emergency physical consultations a specialist in PC went to the emergency area, those who were not attended and needed outpatient consultations were referred to a “palliative care clinic” that would attend them in less than a week. Another limitation found was that the staff from the ES were not familiar with the subcutaneous infusion of opioids such as fentanyl [14,18].

#### 3.3.2. Influence of Palliative Care Nurses in the Emergency Service

In this literature review, one of the objectives was to evaluate the benefit it has on patients, the presence of a nurse specializing in PC in the ES. Some authors have evaluated this fact. Alonso-Babarro et al. [20], developed a study comparing two geographic areas of Madrid, so that one had access to PC services and the other did not. The frequency of hospital death was significantly lower among patients in the area of PC services (61% versus 77%, *p* < 0.001). Patients in the area with PC used ES and inpatient services less frequently than those in the area without PC service. (68% versus 79%, *p* = 0.004 and 66 versus 76%, *p* = 0.012, respectively). The mean hospitalization days among patients who died in the home was 7, compared to 17 who died in the hospital (*p* < 0.001). A higher number of emergency visits is related to a higher probability of dying in the hospital [19]. This is also stated by Sutradhar et al. [11] and by Seow et al. [21].

Sutradhar et al. [12], studied the effect of the presence of a PC home nurse and affirmed that the inclusion of nursing home care palliative care decreased the rate of visits of low gravity to ES (relative rate = 0.53, at a confidence interval of 95% = to 0.50–0.56) and was significantly associated with a greater decrease in the rate of visits to the emergency department in more serious processes (relative rate = 0.37, confidence interval 95% = 0.5–0.38. This fact is affirmed by the authors Seow et al. [21], who developed a study published in 2014 in Ontario (Canada) and who studied these same variables in patients assigned to 11 PC teams that performed scheduled and emergency visits to homes. A high number of emergency visits, 19%, could be avoided with good control of symptoms at home [13].

In the study conducted by Weiland et al. [19], with participants including 444 doctors and 237 nurses working in different hospitals in Australia, it was found that 80% of them had access to specialized PC services (limited to standard working hours) and had received PC training. Moreover, 35.1% reported having a PC unit for hospitalized patients with available beds, 14.7% did not have access or referral to PC services, which prevented optimal care for terminal patients and increased frustration at not being able to provide the level of care they would like compared to those who had beds in place. Five of the respondents stated that they could not directly access PC beds from the emergency department, but that it had to be from another unit [19]. In the same study, most respondents agreed that overload, lack of time, lack of privacy and noise affected patient care (also increasing their frustration, especially in less experienced patients). 93.3% of respondents agreed that a private space should be allocated for terminal patients [18,19,21]. As a solution, there was a greater integration of PC in the ES for this type of patients [19].

Kitsler et al. conducted a clinical trial in the ES where, according to the medical history of patients who went to the emergency room, when they were terminally ill, they were referred directly to PC (in addition to the emergency room). In the experimental group, there was a greater probability of receiving PC during admission and in a shorter time [22]. Weng et al. [16] implemented a PC model in the emergency area based on the training of the emergency nurses in palliative care, also obtaining good results and improving the care of terminal patients.

## 4. Discussion

Regarding emergency nursing, a large part of the professionals felt comfortable in the management of physical symptoms in patients with terminal illnesses. However, it requires training in communication skills at the end of life and the ethical issues that arise when treating this type of patients [23]. It could be argued that, in providing adequate training in PC to emergency nurses, there is an increase in the quality of care, as well as in the referral or consultation with the service of PC [24]. Another problem that arises is the training of nursing homes in palliative care. Many of those nurses are not trained in PC or they are resistant to changes in their job, and they refer more patients to emergency units [25]. Some authors inform that, in addition to personal training, it would be necessary to rotate the management teams, reduce bureaucracy, and improve communication [26,27,28]. Professionals overestimate oncological diagnoses, underestimating chronic obstructive pulmonary disease and dementia, two of the diseases that require more attention in palliative care. Revels et al. [29] corroborate this lack of knowledge among professionals.

Referring to the model of collaboration, various authors agree that the use of a model of care that integrates the PC to the routine practice in the ES can provide significant benefits to patients, including improvement in the quality of life with measures of optimal comfort, control of symptoms, decreased morbidity and hospitalization periods, as well as a decrease in the frequency of use of emergency services and a decrease in days of hospitalization [30]. The ED-PALS care model is considered innovative and appropriate as it combines the strengths of PC teams, emergency teams and hospitalization teams. According to this model, it is estimated that most patients could be seen in less than one hour after referral to ES, while in other care models the waiting time for admission could be more than four hours and many patients had to wait the next day to be referred to palliative care [14].

Several authors have shown the usefulness of a nurse specialist in PC in the ES, or that the collaboration between the emergency department and PC was positive, as we mentioned above would improve health care. Likewise, the widespread complaint was for lack of time, overload, lack of training or inadequate training with respect to the scope of PC [31,32]. In fact, some authors say that respiratory and gastrointestinal oncological processes can be well managed without using emergency units [33]. There are also studies that inform that patients with dementia and good palliative care attend less to emergency units [34]. Furthermore, other authors say that regular care to nursing homes would improve palliative care for older people and reduce visits to emergency units and longs hospitalizations [35]. 

The main limitation of our study has taken place in the selection process. The literature on palliative care for terminal patients in the ES is very limited. In most cases, data analysis and comparison have had to be done because there is no specific record that separates terminal patients from other patients who go to the emergency room. It is also hard to cover and understand the area of this study with a review taking into account the limited literature.

## 5. Conclusions

As conclusions, nurses in the emergency services showed a lack of training in some aspects of PC. Having nurses’ that specialize in PC in the unit or implementing a tripartite collaboration model would improve the care for terminal patients in emergency services. An increase in training would allow professionals to distinguish which patients need palliative care, not only those with oncological pathology, effective control of signs and symptoms, and a decrease in unnecessary invasive processes. Having specialist nurses or a tripartite collaboration model would provide a comprehensive, higher-quality care, which could reduce the number of intrahospital deaths, their frequency, or the days of hospitalization in terminal patients. 

## Figures and Tables

**Table 1:** Overview of selected studies, basic characteristics and results.

Author Year (Nation)	Design	Sample (N)	Results Aim A (to Describe the Training, Capacitation and Interventions of Registered Nurses that Works in the ES Regarding to PC)	Results Aim B (to Describe the Influence of PC in Visits to ES and to Analyze the PC Access Model When Attending Emergency Services)	Evidence Level and Grade of Recommendation
**Pereira MED et al. [11],** **2017 (Portugal)**	Cross-Sectional Study	83	The most used invasive procedures were: IV channeling, IV medication administration and intravenous therapy, blood extractions. They were performed under medical prescription. Only the usual comfort measures were performed in the service. Only 14.5% of non-essential medication was discontinued. This decision was not recorded in 83 per cent of cases. In 71.1% of cases, agony was not diagnosed. In 2.9% of those that did, it was done without recognizing at least two factors proper to agony. The factors that most helped nurses to recognize imminent death were comatose status (94%) and worsening of dyspnea (20.5%). In the face of imminent death, CPR maneuvers and aspiration of secretions were carried out.		2c/B
**Sutradhar R et al. [12], 2016 (Ontario, Canada)**	Cohort study	113,902		Home PC by nursing to terminal patients decreased the rate of emergency visits. The rate of visits to high-acuity ES (per 100 person-days) was 2.35. This rate was reduced to 0.33 during the time during which PC services were used in the home. The rate of visits to low-acuity ES (per 100 person-days) was 0.531; this rate fell to 0.0831 during the time during which PC services were used in the home.	2b/B
**Alsirafy SA et al. [13], 2016.** **(Saudi Arabia)**	Cohort study	154		19% of visits to the ES were considered avoidable due to poorly controlled symptoms.	2b/B
**Koh MYH et al. [14], 2019 (USA)**	Cohort study	340		A tripartite model of collaboration between PC, ES and hospitalization is advocated. It allows earlier access to the palliative care unit and direct income to this unit and care rooms: Patients who went to the ES and could not be attended by the PC service were referred to the “PC clinics”. Patients who required hospitalization were admitted directly from the ES.	2b/B
**Barbera L et al. [15], 2010 (USA)**	Cohort study	91,561		Proper symptom control would decrease the number of unnecessary visits to the U.S.	2b/B
**Weng TC et al. [16], 2017 (Taiwan)**	Clinical trial	648	Capacity of emergency nurses to perform PC: ○ Prior to the intervention: 64 emergency nurses (68.1) knew how to perform PC. ○ End of intervention: 90 (95.7%) emergency nurses knew how to perform PC Consultation with the PC team in the emergency room: Ability to know when and how to consult the PC team increased to 95.7% in the post-intervention phase.		1a/A
**Shearer FM et al. [17], 2014 (Australia)**	Cross-Sectional Study	66.	It is claimed that more training is needed with regard to PC. Only 9.5% of respondents correctly identified the main causes of death, as cancer was overvalued and other diseases such as dementia, or COPD were undervalued. Regarding the management of physical symptoms: staff showed more confidence in symptom management, but deficiencies in ethical and communication issues.		2c/B
**Russ A et al. [18], 2015. (Australia)**	Cross-Sectional Study	65	All staff were comfortable with managing the symptoms. There is a need to increase PC training, especially in ethical and communication issues. The nurses reflected the existence of legal problems that limit the provision of PC. Causes of death in PC patients: emergency personnel overestimate cancer as a cause of death and underestimate other non-cancerous diagnoses.		2c/B
**Weiland TJ et al. [19], 2015 (Australia)**	Cross-Sectional Study	681	Environmental barriers: most respondents agreed that work overload, lack of privacy, lack of time, and noise affected patient care. 73.6% felt unable to proportional the desired level of care to patients with advanced cancer in the U.S. (for reasons independent of training).	Access to PC in ES: 80% of participants had access to specialized PC services 35.1% reported having a PC unit for hospitalized patients with available beds. 14.7% had no access or reference to external PC services	2c/B
**Alonso-Babarro A at al [20], 2013 (Spain)**	Cross-Sectional Study	200,000		524 patients were studied to determine the place of death. A total of 387 (74%) patients died in the hospital, 90 (17%) died in the Home, 29 (6%) died in one and 18 (3%) died in a nursing home. The frequency of hospital death was significantly lower among patients in the area with Home PC equipment (ECPD) (61% versus 77%, *p* < 0.001). Patients in the area with PC equipment used ES less frequently than those in the area without Home PC equipment (68% versus 79%, *p* = 0.004 and 66 versus 76%, *p* = 0.012, respectively). The mean number of days of hospitalization among patients who died in the home was 7, compared to 17 Days of patients who died in the hospital (*p* <0.001)	2c/B
**Seow H et al. [21], 2014 (Canadá)**	Cohort study	3109		In the exposed group (patients with PC team), 970 (31.2%) were in the hospital and 896 (28.9%) had an ES visit in the last two weeks of life, respectively, compared to 1219 (39.3%) and 1070 (34.5%) in the non-exposed group. Fewer exposed patients than non-exposed patients died in the hospital. Specialized community-based PC teams were effective in reducing emergency room visits and hospital deaths. There is a difficulty in communicating with the PC unit outside working hours	2b/B
**Kitsler EA et al. [22], 2015 (USA)**	Clinical trial	134		The experimental group was referred directly to PC (in addition to the corresponding emergency consultation) by the research team when analyzing its medical history. The control group was only derived if requested by the Attending Physician. 88% of the intervention group received care from the palliative care team during their admission, compared to 18% of the control group. In the experimental group palliative care was received on average about 1.48 days compared to the 2.9 days in the control group.	1a/A
